# SARS-CoV-2 uses metabotropic glutamate receptor subtype 2 as an internalization factor to infect cells

**DOI:** 10.1038/s41421-021-00357-z

**Published:** 2021-12-14

**Authors:** Jinliang Wang, Guan Yang, Xinxin Wang, Zhiyuan Wen, Lei Shuai, Jie Luo, Chong Wang, Ziruo Sun, Renqiang Liu, Jinying Ge, Xijun He, Ronghong Hua, Xijun Wang, Xiao Yang, Weiye Chen, Gongxun Zhong, Zhigao Bu

**Affiliations:** 1 State Key Laboratory of Veterinary Biotechnology, Harbin Veterinary Research Institute, Chinese Academy of Agricultural Sciences, Harbin, Heilongjiang China; 2 State Key Laboratory of Proteomics, Beijing Proteome Research Center, National Center for Protein Sciences (Beijing), Beijing Institute of Lifeomics, Beijing, China

**Keywords:** Mechanisms of disease, Molecular biology

## Abstract

Severe acute respiratory syndrome coronavirus 2 (SARS-CoV-2) uses angiotensin-converting enzyme 2 (ACE2) as a binding receptor to enter cells via clathrin-mediated endocytosis (CME). However, receptors involved in other steps of SARS-CoV-2 infection remain largely unknown. Here, we found that metabotropic glutamate receptor subtype 2 (mGluR2) is an internalization factor for SARS-CoV-2. Our results show that mGluR2 directly interacts with the SARS-CoV-2 spike protein and that knockdown of mGluR2 decreases internalization of SARS-CoV-2 but not cell binding. Further, mGluR2 is uncovered to cooperate with ACE2 to facilitate SARS-CoV-2 internalization through CME and mGluR2 knockout in mice abolished SARS-CoV-2 infection in the nasal turbinates and significantly reduced viral infection in the lungs. Notably, mGluR2 is also important for SARS-CoV spike protein- and Middle East respiratory syndrome coronavirus spike protein-mediated internalization. Thus, our study identifies a novel internalization factor used by SARS-CoV-2 and opens a new door for antiviral development against coronavirus infection.

## Introduction

A novel coronavirus named severe acute respiratory syndrome coronavirus 2 (SARS-CoV-2) was identified as the causative pathogen of coronavirus disease 2019 (COVID-19)^1^. SARS-CoV-2 is an enveloped, non-segmented positive-strand RNA virus, belonging to the coronaviridae family, which consists of four genera: *alphacoronavirus, betacoronavirus, gammacoronavirus*, and *deltacoronavirus*^2^. Human coronaviruses usually cause mild respiratory tract infections; however, severe acute respiratory syndrome coronavirus (SARS-CoV), Middle East respiratory syndrome coronavirus (MERS-CoV), and SARS-CoV-2, which all belong to the genus *betacoronavirus*^3^, can cause severe disease and death in humans.

Receptors play key roles in the process of virus infection. Numerous viruses are known to use more than one type of receptor, either in parallel or in series^4,5^. Viruses that undergo rapid mutation can switch receptors^6,7^ or adapt to use alternative receptors when their primary receptor is absent^8^. SARS-CoV-2 is prone to mutations during replication, and many mutations have been reported around the world^9^. Angiotensin-converting enzyme 2 (ACE2) has been well documented as a receptor for SARS-CoV-2, which directly interacts with the SARS-CoV-2 spike (S) protein and renders cells and mice susceptible to SARS-CoV-2 infection^1,10–12^. However, it remains largely unclear whether SARS-CoV-2 also uses other receptors.

Recent studies have identified several proteins or non-protein molecules that facilitate SARS-CoV-2 entry into cells. Heparan sulfate, dendritic cell (DC)-specific intercellular adhesion molecule-3-grabbing non-integrin, and liver/lymph node-specific intracellular adhesion molecules-3 grabbing non-integrin have been reported to serve as auxiliary attachment receptors to promote SARS-CoV-2 infection^13,14^. Neuropilin 1, which can promote SARS-CoV-2 infection in cells that express low levels of ACE2, CD4, and HDL scavenger receptor B type 1 (SR-B1) have also been shown to facilitate SARS-CoV-2 entry, although SR-B1 does not directly interact with the SARS-CoV-2 S protein^15–18^. Transmembrane serine protease 2 (TMPRSS2) cleaves the SARS-CoV-2 S2 subdomain at the S2’ cleavage site to expose the fusion peptide of S protein and initiates membrane fusion^11,19^. Tyrosine-protein kinase receptor UFO (AXL), CD147, Kringle-containing protein marking the eye and the nose protein 1 (KREMEN1), and asialoglycoprotein receptor 1 (ASGR1) have also been reported to promote SARS-CoV-2 infection, and may be potential receptors used by SARS-CoV-2^20–22^, although Bohan et al.^23^ recently reported that AXL directly interacts with virion-associated phosphatidylserine, but not with the SARS-CoV-2 S protein, and cooperates with ACE2 to mediate SARS-CoV-2 attachment and entry. A study by Shilts et al.^24^ reported that CD147 does not interact with the SARS-CoV-2 S protein and has no effect on SARS-CoV-2 entry. All of these cellular factors were identified in vitro, and it is not clear whether the knockout of these genes would affect SARS-CoV-2 replication in vivo.

In this study, we found that metabotropic glutamate receptor 2 (mGluR2) interacts with the SARS-CoV-2 S protein directly and is important for SARS-CoV-2 internalization. mGluR2 is a seven-transmembrane domain receptor and is thought to be functionally involved in cognitive disorders, drug addiction, psychosis, schizophrenia, anxiety, cerebral ischemia, and epilepsy^25–28^. mGluR2 is known to facilitate rabies virus to enter cells^29^. In the present study, we found that knockout of mGluR2 in mice abolished SARS-CoV-2 infection in the nasal turbinates and significantly reduced viral infection in the lungs. Our study suggests that mGluR2 is an internalization factor for SARS-CoV-2 and opens a new door for antiviral development against SARS-CoV-2 infection.

## Results

### mGluR2 is required for SARS-CoV-2 infection and directly interacts with the S protein

**Fig. 1:**
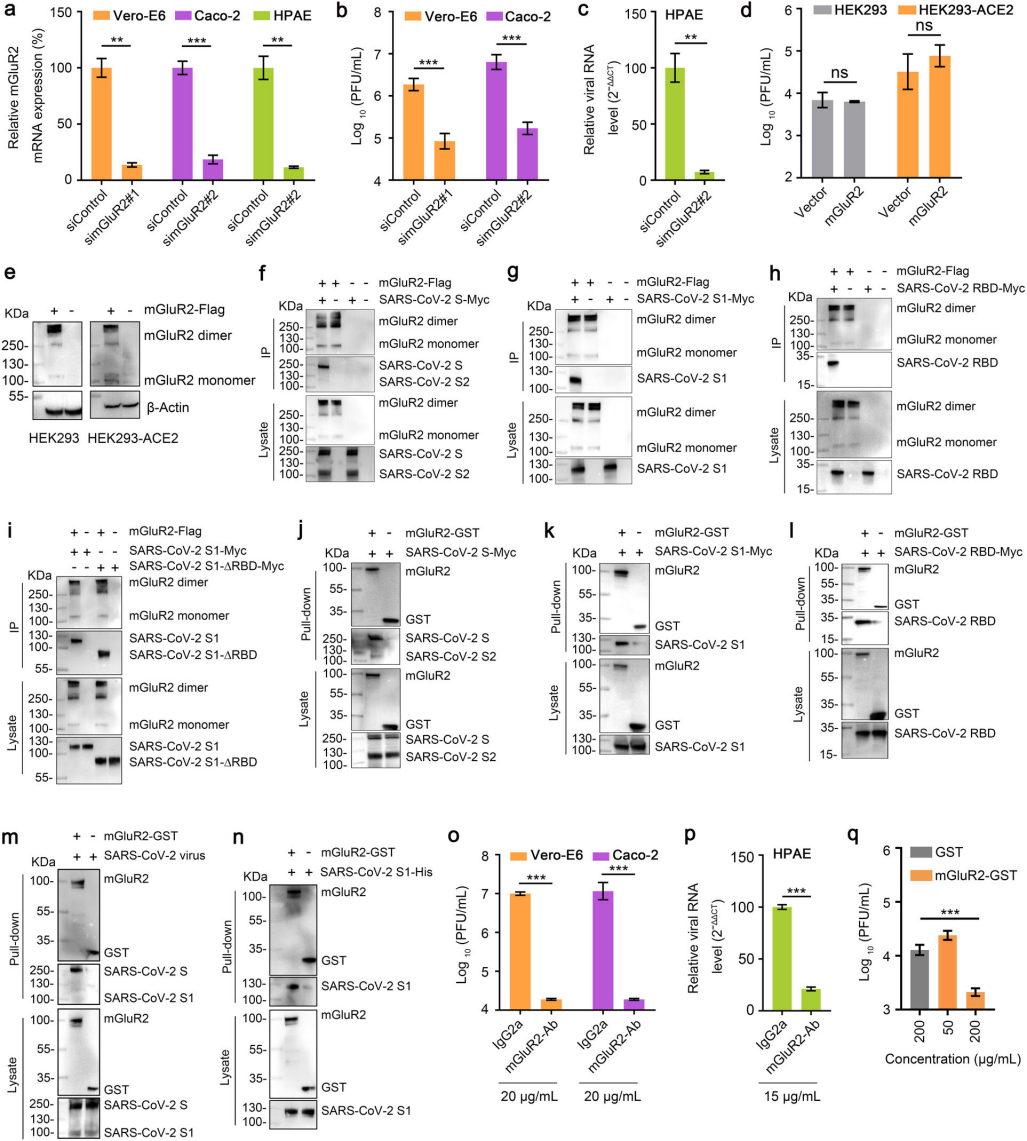
mGluR2 is required for SARS-CoV-2 infection and directly interacts with the S protein. **a** Knockdown of mGluR2 was measured by use of qPCR in Vero-E6 cells, Caco-2 cells, and HPAE cells. simGluR2#1 and simGluR2#2, siRNAs specific for mGluR2 mRNA from monkey and human, respectively. siControl, scrambled RNA. **b**, **c** mGluR2-silenced Vero-E6 cells (**b**), Caco-2 cells (**b**) or HPAE cells (**c**) were infected with HRB25. At 24 h p.i., virus in the culture supernatant or in the cell lysate (HPAE cells) was detected by use of plaque assays or qPCR. **d**, **e** mGluR2-transfected cells were infected with HRB25. At 24 h p.i., virus in the culture supernatant was detected by use of plaque assays (**d**). The expression of exogenous mGluR2 in HEK293 cells and HEK293-ACE2 cells was confirmed by western blotting assay (**e**). **f**–**i** mGluR2-Flag and SARS-CoV-2 S-Myc (**f**), SARS-CoV-2 S1-Myc (**g**), SARS-CoV-2 RBD-Myc (**h**), or SARS-CoV-2 S1-∆RBD-Myc (**i**) were co-transfected into HEK293 cells and then immunoprecipitated by using anti-Flag agarose beads. **j**–**l** Purified recombinant GST-tagged mGluR2 ectodomain (mGluR2-GST) was pooled with lysate from SARS-CoV-2 S-Myc- (**j**), SARS-CoV-2 S1-Myc- (**k**) or SARS-CoV-2 RBD-Myc- (**l**) transfected HEK293 cells and then pulled down by using anti-GST beads. **m**, **n** mGluR2-GST was pooled with cell lysate from SARS-CoV-2-infected Vero-E6 cells (**m**), or purified SARS-CoV-2 S1 subdomain protein (SARS-CoV-2 S1-His) (**n**) and then pulled down by using anti-GST beads. **o**, **p** Vero-E6 cells (**o**), Caco-2 cells (**o**), and HPAE cells (**p**) were treated with mGluR2-Ab at different concentrations or IgG2a (20 μg/mL for Vero-E6 cells and Caco-2 cells; 15 μg/mL for HPAE cells) for 1 h at 4 °C, and then infected with HRB25. Virus in the culture supernatant or in the cell lysate (HPAE cells) was detected by use of plaque assays or qPCR at 48 h p.i.. **q** HRB25 was pooled with different concentrations of mGluR2-GST or GST (200 μg/mL), and then Vero-E6 cells were infected by mixing for 1 h at 37 °C. Virus in the culture supernatant was detected by use of plaque assays at 24 h p.i.. The data shown are representative results from three independent experiments (**a**–**d**, **o**–**q** *n* = 3), means ± SD, Student’s *t*-test, ns, not significant, **P < 0.01, ***P < 0.001.

To test whether mGluR2 is needed for SARS-CoV-2 infection, we first tested mGluR2 expression in Vero-E6 cells and Caco-2 cells, which are susceptible to SARS-CoV-2 infection. mGluR2 was labeled on the membrane of the two cell lines and examined by using flow cytometry. The results showed that both cell lines expressed mGluR2 on the cell surface (Supplementary Fig. S1a, b). We then knocked down mGluR2 expression by transfecting Vero-E6 cells and Caco-2 cells with the specific siRNA to mGluR2 mRNA. Compared with cells mock-transfected with scrambled siRNA, the expression of mGluR2 mRNA was significantly reduced in both cell types at 18 h post-transfection (Fig. 1a). At 72 h post-transfection, Vero-E6 cells and Caco-2 cells were each infected with the SARS-CoV-2 human isolate SARS-CoV-2/HRB25/human/2020/CHN (HRB25). Virus released into the culture supernatants by infected cells was detected by viral titration at 24 h post-infection (p.i.). Knockdown of mGluR2 significantly decreased the viral titers in both cells (Fig. 1b). mGluR2 expression was also found on the surface of primary human pulmonary alveolar epithelial cells (HPAE cells) (Supplementary Fig. S1c), and knockdown of mGluR2 significantly decreased the viral RNA level in the cell lysate (Fig. 1a, c). We further tested whether mGluR2 overexpression facilitates SARS-CoV-2 infection by transfecting mGluR2 cDNA into HEK293 cells that express ACE2 at a low level and a stable HEK293 cell line expressing human ACE2 (HEK293-ACE2) (Supplementary Fig. S1d). The overexpression of mGluR2 was confirmed by western blotting at 48 h post-transfection (Fig. 1e). The cells were infected with HRB25 at 48 h post-transfection. Virus in the supernatants was detected by viral titration at 24 h p.i.. The viral titer in mGluR2 cDNA-transfected cells was comparable to that of vector-transfected cells for both cell lines (Fig. 1d), indicating that mGluR2 overexpression has no effect on SARS-CoV-2 infection. These results suggest that mGluR2 is required for SARS-CoV-2 infection.

The first step in coronavirus infection is binding to cellular receptors via the viral envelope S protein^3^. The S protein is composed of the S1 and S2 subdomains. The S1 subdomain contains the receptor-binding domain (RBD) and is responsible for binding to specific receptors. The S2 subdomain contains a fusion peptide and is responsible for the fusion of the viral membrane with the cellular membrane^3,30^. We next used co-immunoprecipitation assays to test whether mGluR2 interacts with the SARS-CoV-2 S protein. Flag-tagged mGluR2 (mGluR2-Flag) was co-expressed with Myc-tagged S protein (S-Myc) in plasmid-transfected HEK293 cells. Immunoblotting for S-Myc demonstrated that mGluR2 interacts with the SARS-CoV-2 S protein specifically (Fig. 1f). To characterize the domain of the SARS-CoV-2 S protein that interacts with mGluR2, we performed a co-immunoprecipitation analysis using the S1 subdomain and the RBD of the SARS-CoV-2 S protein in plasmid-transfected HEK293 cells. We found that both the S1 and RBD interacted with mGluR2 (Fig. 1g, h). To test whether mGluR2 interacts with regions other than the RBD in the S1 subdomain, we performed a co-immunoprecipitation assay using mGluR2 and truncated S1 without the RBD (S1-∆RBD) in plasmid-transfected HEK293 cells. The results showed that mGluR2 also interacts with S1-∆RBD (Fig. 1i). We then performed pull-down assays to determine whether mGluR2 interacts with the SARS-CoV-2 S protein directly. Purified recombinant GST-tagged ectodomain of mGluR2 (mGluR2-GST) was pooled with lysate from HEK293 cells transfected with the SARS-CoV-2 S protein, S1 subdomain, or RBD, respectively. We found that purified mGluR2-GST successfully pulled down the SARS-CoV-2 S protein, S1 subdomain, and RBD (Fig. 1j–l), demonstrating that the ectodomain of mGluR2 interacts with the SARS-CoV-2 S protein. We also performed a pull-down assay by using the purified mGluR2-GST protein and HRB25-infected Vero-E6 cell lysate and found that mGluR2-GST pulled down the SARS-CoV-2 S protein from virus-infected cell lysate (Fig. 1m). Purified mGluR2-GST and SARS-CoV-2 S1 subdomain protein (SARS-CoV-2 S1-His) were subjected to pull-down assay. We found that mGluR2-GST successfully pulled down SARS-CoV-2 S1-His (Fig. 1n). These results indicate that the ectodomain of mGluR2 directly interacts with the SARS-CoV-2 S protein.

To investigate whether the ectodomain of mGluR2 is important for SARS-CoV-2 infection, we tested whether an antibody to the ectodomain of mGluR2 could block SARS-CoV-2 infection in vitro. Vero-E6 cells and Caco-2 cells were treated with the mGluR2 antibody at indicated concentrations for 1 h at 4 °C, and then incubated with HRB25. Infectious titers in the supernatant were detected by virus titration at 48 h p.i.. The results showed that the mGluR2 antibody efficiently inhibited SARS-CoV-2 infection in Vero-E6 and Caco-2 cells (Fig. 1o). The cytotoxicity of the mGluR2 antibody in both cell types was also tested, and the results confirmed that the cell viability was unaffected at the concentration used (Supplementary Fig. S2). An antibody against the ectodomain of ACE2 was used as a control, which efficiently inhibited SARS-CoV-2 infection in Vero-E6 and Caco-2 cells and that cell viability was unaffected (Supplementary Fig. S3a, b). We confirmed the results using HPAE cells and again found that the mGluR2 antibody was not cytotoxic at the concentration used (Supplementary Fig. S2) and that it significantly decreased the viral RNA level in the cell lysate (Fig. 1p).

We then tested whether mGluR2 could block SARS-CoV-2 infection in vitro. SARS-CoV-2 was pooled with different concentrations of mGluR2-GST for 1 h at 4 °C, and then Vero-E6 cells were incubated with the mixtures for 1 h at 37 °C. The infectious titers in the supernatants of the infected cells were detected at 24 h p.i. mGluR2-GST showed an inhibitory effect in a dose-dependent manner (Fig. 1q). Together, these results suggest that mGluR2 is an important host factor for SARS-CoV-2 infection.

### SARS-CoV-2 internalization requires mGluR2

**Fig. 2:**
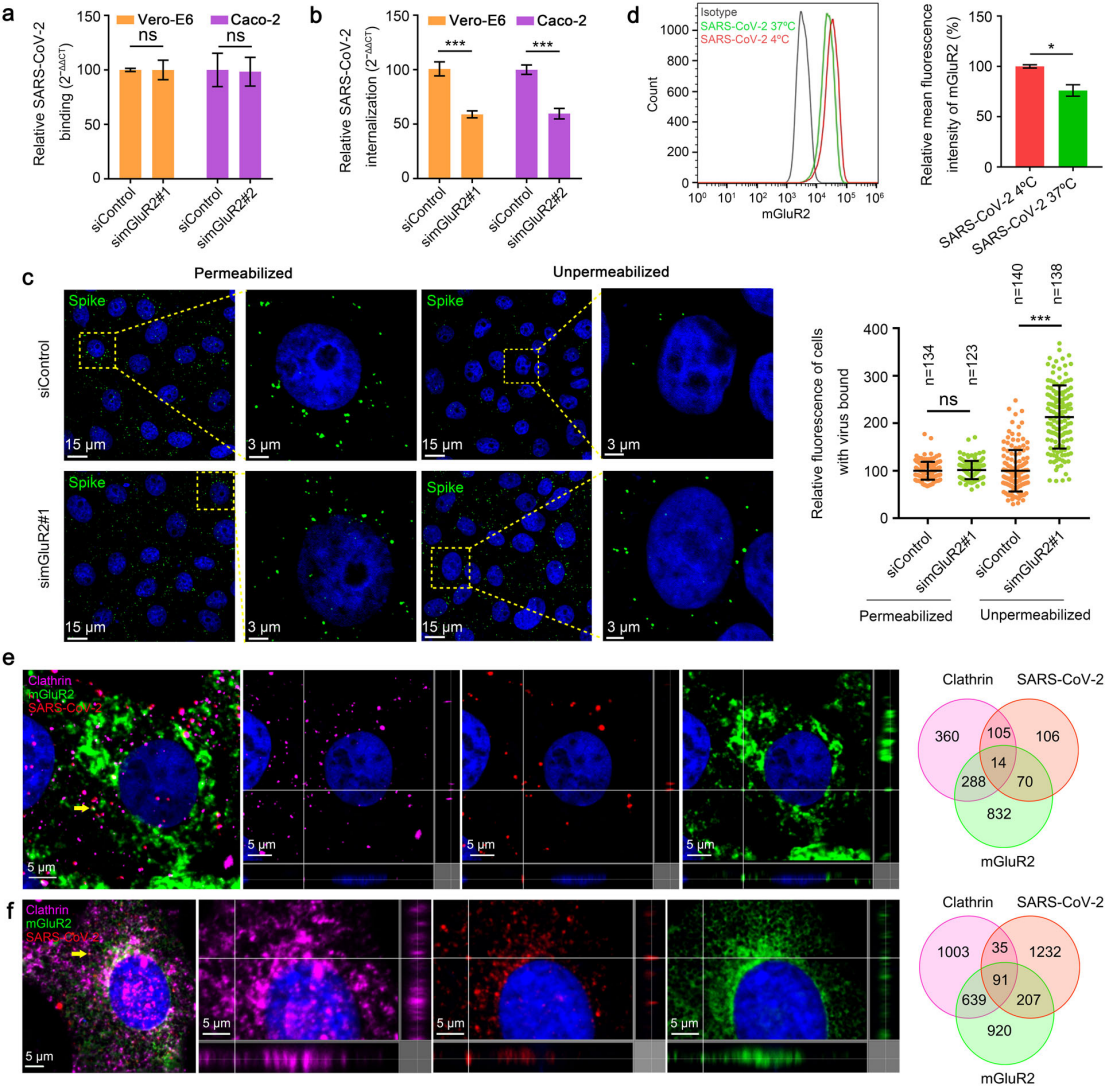
mGluR2 is required for internalization of SARS-CoV-2 but not for cell binding. **a**, **b** SARS-CoV-2 binding (**a**) and internalization (**b**) assays were performed in mGluR2-silenced Vero-E6 cells and Caco-2 cells. Viral binding or internalization was quantified by normalization to the respective scrambled siRNA-transfected cells. **c** Vero-E6 cells were treated as described in **a**, except they were not treated with acid buffer/trypsin. Cell nuclei (blue), SARS-CoV-2 S protein (green). Left: representative images are shown, and the dashed box is magnified at the indicated location of the same image. Right: the fluorescence signal of SARS-CoV-2 particles was quantified by immunofluorescence staining using ZEN software. The relative fluorescence of cell-bound HRB25 under permeabilized or unpermeabilized conditions was quantified by normalization to the corresponding scrambled siRNA-transfected cells. The circles represent individual data points. *n*, the number of quantified cells. Data represent the sum of three independent experiments. **d** Surface expression level of mGluR2 was detected by using flow cytometry after infection with HRB25 at 37 °C for 30 min under unpermeabilized conditions in Vero-E6 cells. **e**, **f** Multiplex immunofluorescence was performed in Vero-E6 cells (**e**) and HPAE cells (**f**). Colocalization of mGluR2 (green), SARS-CoV-2 N protein (red), and clathrin (purple) was observed and quantified. The yellow arrows indicate the representative colocalization of mGluR2 (green), SARS-CoV-2 N protein (red), and clathrin (purple), shown in three dimensions. The data shown are representative results from three independent experiments (**a**, **b**, **d** *n* = 3), means ± SD, Student’s *t*-test, ns, not significant, *P < 0.05, ***P < 0.001.

We next tested which stage of SARS-CoV-2 entry involves mGluR2. We performed mGluR2 RNAi assays to determine whether knocking down mGluR2 expression affected the binding or internalization of SARS-CoV-2. mGluR2-silenced Vero-E6 cells, Caco-2 cells, and control cells were incubated with HRB25 at 4 °C for 1 h and washed to remove unbound virus. Then the cells were shifted to 37 °C for 1 h to allow the internalization of bound viruses. The cells were washed with normal PBS or acid buffer/trypsin, which could efficiently remove cell surface-bound SARS-CoV-2 on both cell lines (Supplementary Fig. S4a, b). The washed cells were lysed for quantitative real-time polymerase chain reaction (qPCR) detection of SARS-CoV-2 that was bound to the cell surface or had entered the cells. We found that the amount of virus bound on the mGluR2-silenced cells was comparable to that on the control cells; however, significantly fewer viruses entered the mGluR2-silenced cells than the control cells (Fig. 2a, b), indicating that mGluR2 silencing affected the internalization of SARS-CoV-2 while having no effect on binding to cells.

To verify these results, we performed a microscopy-based assay to monitor the internalization of SARS-CoV-2. The cells were incubated under unpermeabilized or permeabilized conditions with an antibody against the SARS-CoV-2 S protein, and then stained to visualize the viral particles. The fluorescence intensity of each cell was then calculated. The fluorescence intensity under unpermeabilized conditions indicates the viral particles that were unable to enter the cells, whereas that under permeabilized conditions indicates the total number of viral particles. We found that the fluorescence intensity in mGluR2-silenced Vero-E6 cells was significantly higher than that in scrambled siRNA-transfected cells under unpermeabilized conditions, but similar to that of scrambled siRNA-transfected cells under permeabilized conditions (Fig. 2c). These results confirm that mGluR2 is required for the internalization of SARS-CoV-2 but not cell binding.

We next investigated whether mGluR2 is internalized with SARS-CoV-2. The expression of mGluR2 on the cell surface before and after infection with SARS-CoV-2 was first quantitatively determined by flow cytometry under unpermeabilized conditions. The results showed that SARS-CoV-2 infection leads to a significant decrease in the cell surface expression of mGluR2 (Fig. 2d), indicating that mGluR2 is internalized upon infection. Because it has been suggested that SARS-CoV-2 can enter host cells through CME^31^, we next examined the subcellular localization of SARS-CoV-2 particles, mGluR2, and clathrin by using multiplex immunofluorescence staining in HRB25-infected Vero-E6 cells and HPAE cells. We found that SARS-CoV-2 particles, mGluR2, and clathrin co-localized in infected cells (Fig. 2e, f), indicating that the SARS-CoV-2-mGluR2 complex internalizes together.

### mGluR2 interacts with ACE2

**Fig. 3:**
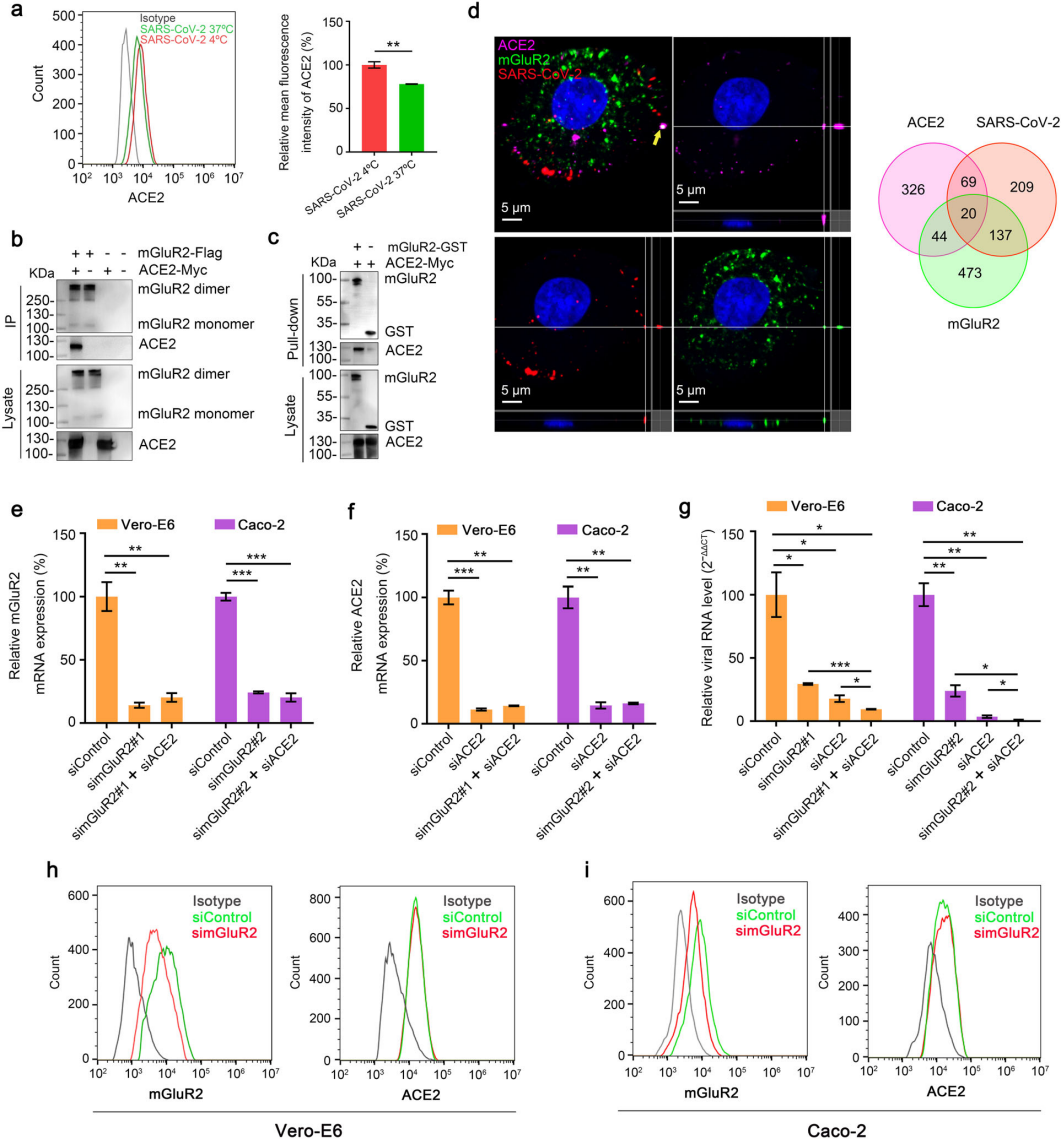
mGluR2 interacts with ACE2. **a** The surface expression level of ACE2 was detected by using flow cytometry after infection with HRB25 at 37 °C for 30 min under unpermeabilized conditions in Vero-E6 cells. **b** HEK293 cells were co-transfected with mGluR2-Flag and ACE2-Myc. Cell lysates were immunoprecipitated by using anti-Flag agarose beads. **c** mGluR2-GST was pooled with lysate from ACE2-Myc-transfected HEK293 cells and then pulled down by using anti-GST beads. **d** Multiplex immunofluorescence was performed in Vero-E6 cells. Colocalization of mGluR2 (green), SARS-CoV-2 N protein (red), and ACE2 (purple) was observed and quantified. The yellow arrowhead indicates the representative colocalization of mGluR2 (green), SARS-CoV-2 N protein (red), and ACE2 (purple), shown in three dimensions. **e**, **f** Knockdown of mGluR2 (**e**) or ACE2 (**f**) was measured by use of qPCR in Vero-E6 cells and Caco-2 cells. simGluR2#1 and simGluR2#2, siRNAs specific for mGluR2 mRNA from monkey and human, respectively; siACE2, siRNA specific for ACE2; siControl, scrambled RNA. **g** mGluR2-silenced, ACE2-silenced Vero-E6 cells, or Caco-2 cells were infected with HRB25. At 24 h p.i., virus in the cell lysate was detected by use of qPCR. **h**, **i** The cell surface expression of mGluR2 or ACE2 in mGluR2-silenced Vero-E6 cells (**h**) or Caco-2 cells (**i**) was detected by flow cytometry. The data shown are representative results from three independent experiments (**a**, **e**–**g** *n* = 3), means ± SD, Student’s *t*-test, *P < 0.05, **P < 0.01, ***P < 0.001.

ACE2 is now well characterized as a receptor for SARS-CoV-2^1,12^. We have shown that SARS-CoV-2 infection also affects the cell surface expression of ACE2 (Fig. 3a), which indicates that ACE2 internalizes with SARS-CoV-2. Therefore, ACE2 might cooperate with mGluR2 to mediate internalization. A direct interaction between mGluR2 and ACE2 would strongly support the hypothesis. The result from co-immunoprecipitation assays revealed that mGluR2 interacts with ACE2 (Fig. 3b), and pull-down assays demonstrated that the ectodomain of mGluR2 interacts with ACE2 (Fig. 3c). We further performed multiplex immunofluorescence to detect SARS-CoV-2, ACE2, and mGluR2 in HRB25-infected Vero-E6 cells. The results showed that ACE2, SARS-CoV-2, and mGluR2 co-localized in cells (Fig. 3d). We further performed RNAi assays in Vero-E6 cells and Caco-2 cells to test whether knockdown of mGluR2 and ACE2 has a synergistic inhibitory effect on SARS-CoV-2 infection. The results showed that knockdown of mGluR2 together with ACE2 led to significantly lower levels of viral RNA than knockdown of mGluR2 or ACE2 alone at 24 h p.i. (Fig. 3e–g). We also demonstrated that knockdown of mGluR2 had no effect on the cell surface expression of ACE2 in both cell types (Fig. 3h, i).

### mGluR2 is expressed in the respiratory system

**Fig. 4:**
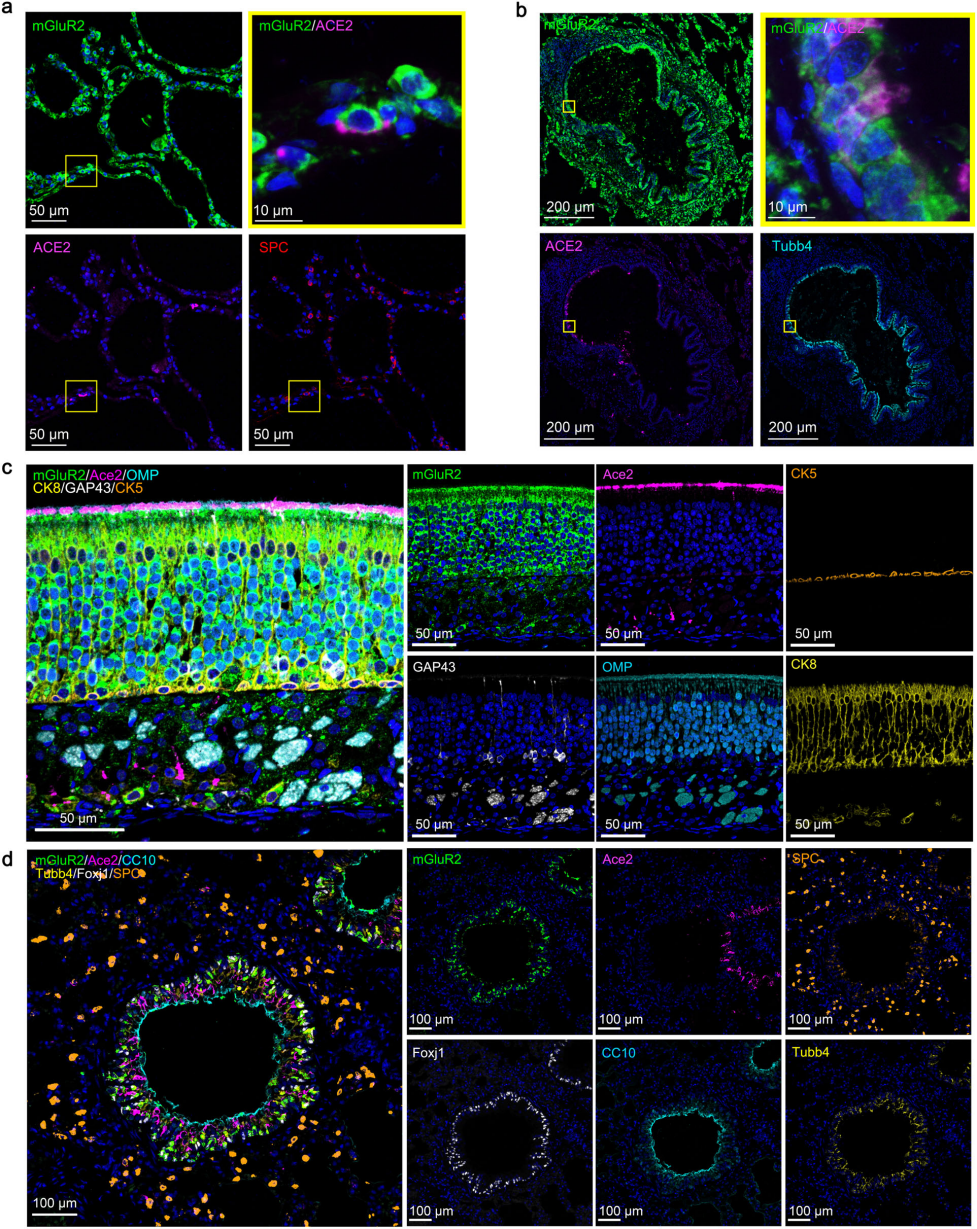
mGluR2 is expressed in the respiratory system. **a**, **b** Multiplex immunofluorescence staining for the detection of mGluR2-positive cells in a normal human lung section. mGluR2 (green), ACE2 (magenta), Tubb4 (cyan), and SPC (red). The yellow areas are shown adjacently at a higher magnification in the alveoli (**a**) or bronchia (**b**). **c** Multiplex immunofluorescence staining for the detection of mGluR2-positive cells in olfactory epithelium sections of young mouse. mGluR2 (green), Ace2 (magenta), GAP43 (white), CK5 (gold), CK8 (yellow), and OMP (cyan). **d** Multiplex immunofluorescence staining for the detection of mGluR2-positive cells in lung sections of young mouse. mGluR2 (green), Ace2 (magenta), Foxj1 (white), SPC (gold), Tubb4 (yellow), and CC10 (cyan).

mGluR2 is known to be expressed mainly in neurons^32^. To investigate the distribution of mGluR2 in the cells of the respiratory tract, we performed multiplex immunofluorescence staining on normal human lung sections, mouse turbinate sections, and mouse lung sections. In human lung, the mGluR2 expression was found in SPC^+^ alveolar type II cells and Tubb4^+^ ciliated cells; ACE2 expression was also detected in these cells (Fig. 4a, b). In nasal turbinates of mice, relatively high mGluR2 expression was detected in CK8^+^ sustentacular cells and OMP^+^GAP43^+^ olfactory neurons, whereas Ace2 expression was limited to CK8^+^ cells in the olfactory epithelium (Fig. 4c). In the lungs of mice, mGluR2 was mainly expressed in CC10^+^ club cells, SPC^+^ alveolar type II cells, and Tubb4^+^Foxj1^+^ ciliated cells (Fig. 4d).

### mGluR2 is important for SARS-CoV-2 to infect mice

**Fig. 5:**
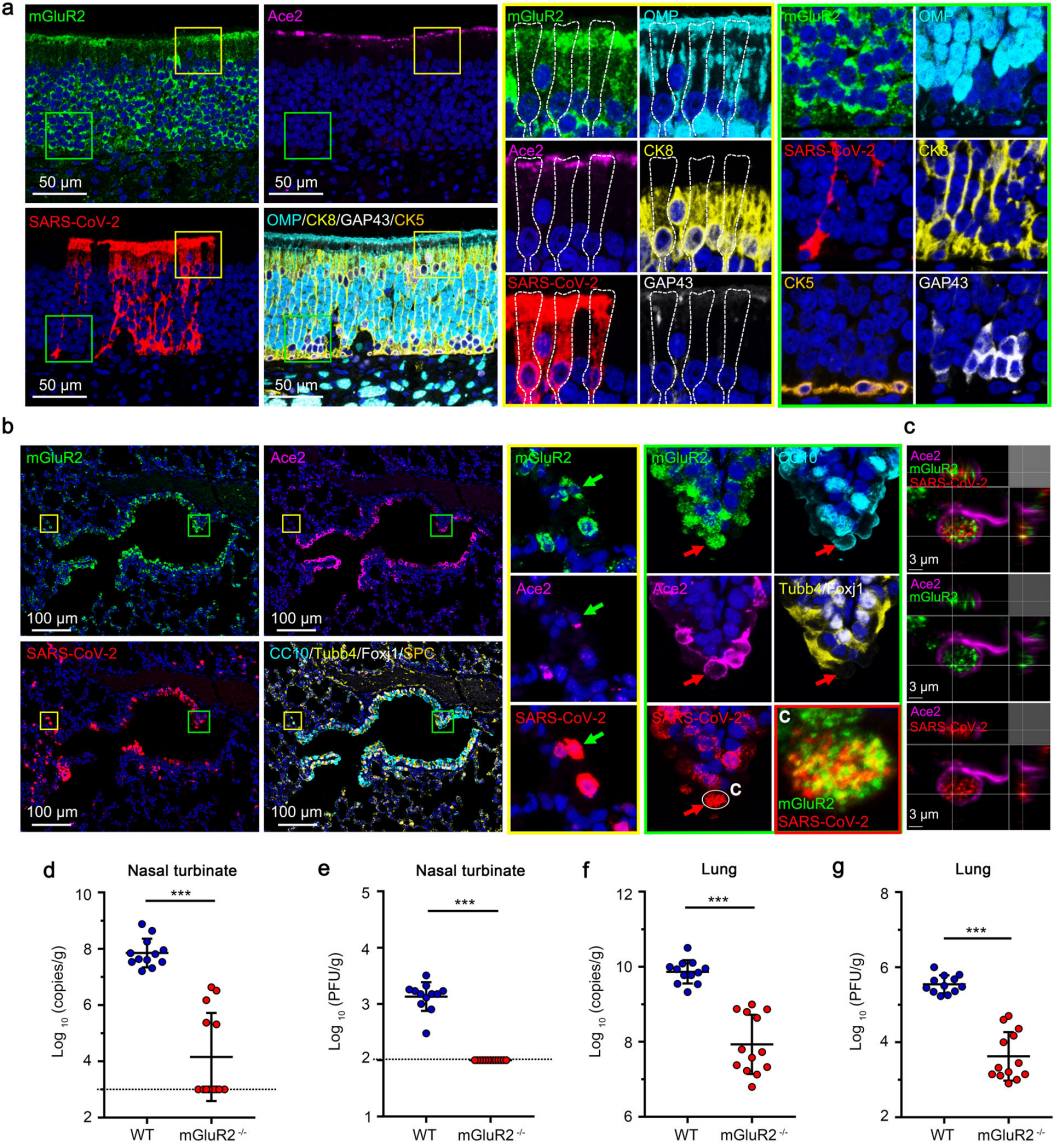
mGluR2 is important for SARS-CoV-2 infection of mice. **a** Multiplex immunofluorescence staining for the detection of mGluR2-positive cells in olfactory epithelium sections of young mouse infected with SARS-CoV-2 on day 3 p.i.. SARS-CoV-2 N protein (red), mGluR2 (green), Ace2 (magenta), GAP43 (white), CK5 (gold), CK8 (yellow), and OMP (cyan). The yellow and green areas are shown adjacently at a higher magnification, respectively. The dashed box indicates the individual cell boundary. **b** Multiplex immunofluorescence staining for the detection of mGluR2-positive cells in lung sections of young mouse infected with SARS-CoV-2 on day 3 p.i.. SARS-CoV-2 N protein (red), mGluR2 (green), Ace2 (magenta), Foxj1 (white), SPC (gold), Tubb4 (yellow), and CC10 (cyan). The yellow and green areas are shown adjacently at a higher magnification, respectively. The red arrows indicate a mGluR2^+^/SARS-CoV-2^+^/Ace2^+^/CC10^+^ cell, and the green arrows indicate a mGluR2^+^/SARS-CoV-2^+^/Ace2^+^ cell. **c** The 3D-rendered image was generated by using Imaris software and the co-localization of mGluR2 and SARS-CoV-2 from the two single fluorescence channels is showed. **d**–**g** mGluR2 gene knockout (mGluR2^*−/−*^) (*n* = 13) and WT (*n* = 12) mice were infected with HRB26M (150 PFU/mouse), a mouse-adapted SARS-CoV-2 strain, via intranasal inoculation. At 3 days p.i., viral RNA copies and virus titers in the nasal turbinates (**d**, **e**) and lungs (**f**, **g**) were determined by use of qPCR (**d**, **f**) and plaque assays (**e**, **g**), respectively. The horizontal dashed line indicates the limit of detection. Data represent the sum of three independent experiments (**d**–**g**), means ± SD, Student’s *t*-test, ***P < 0.001.

We performed additional multiplex immunofluorescence staining on turbinate sections and lung sections of SARS-CoV-2-infected mice, and found that the SARS-CoV-2-infected cells were mainly mGluR2^+^ cells. In the nasal turbinates of mice, high levels of SARS-CoV-2 N protein and mGluR2 expression were detected in CK8^+^ sustentacular cells in the olfactory epithelium (Fig. 5a). In the lungs, colocalization of SARS-CoV-2 and mGluR2 was readily observed in some of the SARS-CoV-2-infected Ace2^+^ cells (Fig. 5b, c). These results indicate that mGluR2 is widely expressed in the cells of the respiratory tract that could be infected by SARS-CoV-2.

We next asked whether mGluR2 is important for SARS-CoV-2 infection in vivo. mGluR2 knockout (mGluR2^*−/−*^) mice were generated by using the CRISPR/Cas9 system (Supplementary Fig. S5a) and their genotype was identified with PCR (Supplementary Fig. S5b). Twelve wild-type (WT) mice and 13 mGluR2^*−/−*^ mice were intranasally inoculated with 150 plaque-forming units (PFU) of the mouse-adapted HRM26M virus^33^. The viral RNA and infectious viruses in the nasal turbinates and lungs were detected at 3 days p.i. by use of qPCR and viral titration. Compared with WT mice, mGluR2^*−/−*^ mice showed significantly decreased viral RNA copies in their nasal turbinates and lungs (Fig. 5d, f). Infectious virus was not detected in the nasal turbinates of mGluR2^*−/−*^ mice (Fig. 5e) and the level of infectious virus was 100-times lower in the lungs of mGluR2^*−/−*^ mice than that of WT mice (Fig. 5g).

### Betacoronavirus internalization requires mGluR2

SARS-CoV and MERS-CoV are also highly pathogenic coronaviruses to humans^3^. We, therefore, asked whether mGluR2 is important for SARS-CoV and MERS-CoV to enter cells. We first investigated whether the S proteins of SARS-CoV and MERS-CoV interacted with mGluR2. Co-immunoprecipitation assays demonstrated that SARS-CoV S interacts with mGluR2, whereas MERS-CoV S does not interact with mGluR2 (Supplementary Fig. S6a, b). DPP4, a known receptor of MERS-CoV^34^, interacted with the ectodomain of mGluR2 (Supplementary Fig. S6c, d). To evaluate infection, we used recombinant vesicular stomatitis viruses (VSV) expressing SARS-CoV S (rVSV-SARS-CoV-S) or MERS-CoV S (rVSV-MERS-CoV-S) to substitute for authentic SARS-CoV and MERS-CoV, respectively. These two chimeric VSVs were generated following the previously described strategy^35^, in which the open reading frame (ORF) of the G gene is replaced with the ORF of the SARS-CoV or MERS-CoV S gene. Antibodies against mGluR2 efficiently blocked the infection of rVSV-SARS-CoV-S and rVSV-MERS-CoV-S in Vero-E6 cells (Supplementary Fig. S6f), but failed to block the infection of VSV in Vero-E6 cells (Supplementary Fig. S6e). We next examined the internalization of rVSV-SARS-CoV-S and rVSV-MERS-CoV-S in Vero-E6 cells. The cell surface-bound rVSV-SARS-CoV-S and rVSV-MERS-CoV-S can be efficiently removed by acid buffer/trypsin (Supplementary Fig. S4c, d). mGluR2 knockdown significantly decreased the internalization of both viruses but had no effect on binding to the cells (Supplementary Fig. S6g, h). Together, these results demonstrate that mGluR2 is important for the internalization of SARS-CoV and MERS-CoV.

## Discussion

To our knowledge, our study is the first to demonstrate that mGluR2 is an important host factor for SARS-CoV-2 infection. The results from siRNA silencing, protein interaction, antibody blocking, soluble protein neutralization and image analysis, in addition to experiments with mGluR2 gene knockout mice and immunofluorescence assays, strongly suggest that mGluR2 is an important internalization factor for SARS-CoV-2 infection. The findings that mGluR2 directly interacts with ACE2, that mGluR2, ACE2, and SARS-CoV-2 colocalize in cells, and that knockdown of mGluR2 affects SARS-CoV-2 internalization but not binding indicate that mGluR2 cooperates with ACE2 to mediate SARS-CoV-2 internalization. mGluR2 is also important for SARS-CoV and MERS-CoV S protein-mediated internalization, suggesting that mGluR2 likely plays a conserved role in the internalization of coronaviruses, at least betacoronaviruses. These findings will help promote the development of novel approaches to inhibit coronavirus infection by targeting mGluR2.

There are several possible mechanisms for mGluR2-promoted virus internalization. One possibility is that mGluR2 plays a general role in endocytosis in the cell. However, we found that the uptake of epidermal growth factor (EGF), a typical CME cargo, is unaltered in mGluR2-silenced Vero-E6 cells (Supplementary Fig. S7), which indicates that mGluR2 is unlikely to play a general role in the CME process for all cargos. Further studies are needed to test whether mGluR2 is required for other CME-dependent cargos. Another possibility is that mGluR2 affects the endocytosis of the virus by interacting with the viral glycoprotein and/or its receptor. In this study, we found that mGluR2 interacts with SARS-CoV-2 S, SARS-CoV S, and their receptor ACE2. Of note, we also found that mGluR2 does not interact with MERS-CoV S but interacts with its receptor DPP4. We cannot exclude the possibility that mGluR2 affects viral internalization through CME-independent ways. SARS-CoV-2 can enter cells through direct fusion at the plasma membrane in the presence of TMPRSS2 or through CME following fusion at the endosome that is mediated by endosomal cysteine protease cathepsin L^11,36^. We used camostat mesylate, which inhibits TMPRSS2, or/and E64D, which inhibits cathepsin L, to test whether mGluR2 is required for TMPRSS2-promoted SARS-CoV-2 infection in Caco-2 cells^37,38^. After treatment with camostat mesylate or E64D, mGluR2 silencing significantly decreased SARS-CoV-2 infection of the cells (Supplementary Fig. S8a, b). Our findings indicate that mGluR2 might affect direct fusion during SARS-CoV-2 infection. It will be valuable to explore the details of mGluR2-promoted virus infection in future studies.

We were not able to find or construct a mGluR2-negative SARS-CoV-2 non-susceptible cell line to test whether overexpression of mGluR2 can render a cell line susceptible to SARS-CoV-2 infection, because mGluR2 is widely expressed in different cells (although the expression level varies depending on the cell type), and it is difficult to disrupt the expression of mGluR2 by CRISPR/Cas9 due to the complexity of mGluR2 gene transcription. However, the lack of viral replication in the nasal turbinates of mGluR2 knockout mice clearly demonstrated that mGluR2 is a crucial factor for SARS-CoV-2 infection. mGluR2 gene knockout did not completely prevent SARS-CoV-2 infection in the lungs, suggesting that other entry receptors may also contribute to SARS-CoV-2 infection, which is further supported by recent findings that several different proteins, including AXL, KREMEN1, and ASGR1, also facilitate SARS-CoV-2 entry^20,22^.

Although human coronaviruses are typically associated with respiratory tract diseases, three human coronaviruses have been shown to infect neurons: HCoV-229E, HCoV-OC43, and SARS-CoV^39^. MERS-CoV has also been associated with neurologic disease in some cases^40^. Increasing evidence indicates that SARS-CoV-2 not only affects the respiratory tract but also impacts the central nervous system, resulting in neurologic symptoms, such as loss of smell and taste, headache, and nausea^41–43^. A recent study revealed that SARS-CoV-2 can enter the nervous system by crossing the neural-mucosal interface in the olfactory mucosa^44^, which indicates that the nasal turbinate is an important access point for SARS-CoV-2 to enter the brain. In the present study, we found that mGluR2 is essential for SARS-CoV-2 infection of the nasal turbinate. Given that mGluR2 is widely expressed in neurons, we cannot exclude the possibility that mGluR2 is involved in SARS-CoV-2 neurotropism. Unfortunately, investigation of the role of mGluR2 in SARS-CoV-2 neurotropism is very difficult due to the lack of suitable in vitro or in vivo models. The discovery of a role for mGluR2 in SARS-CoV-2 infection supports the possibility that SARS-CoV-2 could use mGluR2 to enter and infect neuron cells.

## Materials and methods

### Cell lines

HEK293 cells (ATCC, CRL-1573), Caco-2 cells (ATCC; HTB-37), and Vero-E6 cells (ATCC, CRL-1586) were maintained in Dulbecco’s modified Eagle’s medium (DMEM) supplemented with 10% fetal bovine serum (FBS), 1% penicillin/streptomycin, and _L_-glutamine at 37 °C in 5% CO_2_. Human Pulmonary Alveolar Epithelial Cells (HPAE cells) (ScienCell Research Laboratories, 3200) were cultured in alveolar epithelial cell medium (ScienCell Research Laboratories, 3201). HEK293 cells expressing human ACE2 (HEK293-ACE2 cells) were generated by transducing an ACE2-expressing lentiviral vector, and selected with puromycin. After selection, the cells were maintained with puromycin.

### Viruses

SARS-CoV-2/HRB25/human/2020/CHN (HRB25, GISAID access no. EPI_ISL_467430), SARS-CoV-2/HRB26/mouse/2020/CHN (HRB26M, GISAID access no. EPI_ISL_459910), and VSV were maintained in our laboratory. VSV chimeras expressing the S protein of SARS-CoV (rVSV-SARS-CoV-S) or MERS-CoV (rVSV-MERS-CoV-S) were generated as previously described^35^. Virus titers were determined by using standard plaque assays on Vero-E6 cells, and virus stocks were aliquoted and stored at −80 °C until use. All experiments with infectious SARS-CoV-2 were performed in the biosafety level 4 and animal biosafety level 4 facilities in the Harbin Veterinary Research Institute (HVRI) of the Chinese Academy of Agricultural Sciences (CAAS).

### Mice

All mice were bred on the C57BL/6J background. Six- to eight-week-old C57BL/6J mice were obtained from Vital River Laboratories (Vital River Laboratories, Beijing, China). mGluR2^*−/−*^ mice were from the Model Animal Research Center of Nanjing University. mGluR2^*−/−*^ mice were generated by crossing and using the CRISPR/Cas9 system. Cas9 mRNA and sgRNA were co-injected into zygotes. sgRNA (mGluR2-sgRNA 1: CCTCTTACTCCGTGGCATAT, mGluR2-sgRNA 2: TGGGGATGAGAGCTAACACT, mGluR2-sgRNA 3: GCGACCAGATCCCCTAGGTC, mGluR2-sgRNA 4: TGTAGAGTTTAAGGCTCGCC) directed Cas9 endonuclease cleavage upstream of exon 1 and the 3’UTR, creating a double-strand break. Such breaks were repaired by non-homologous end joining, which resulted in the destruction of the mGluR2 gene. Pups were genotyped by use of PCR, followed by sequencing analysis. Mice were maintained under conventional conditions in the HVRI of CAAS, which is approved for such use by the Ministry of Agriculture and Rural Affairs of China. All institutional and national guidelines for the care and use of laboratory animals were followed. All mouse experiments were carried out in strict accordance with the recommendations in the Guide for the Care and Use of Laboratory Animals of the Ministry of Science and Technology of the People’s Republic of China. The protocols were approved by the Committee on the Ethics of Animal Experiments of the HVRI of CAAS.

### Plasmids

pCAGGS-mGluR2-Flag was described previously^29^. Human ACE2 and DPP4 cDNAs were cloned into the pCAGGS-Flag and pCAGGS-Myc vectors as indicated in our study, and confirmed by sequencing analysis. The SARS-CoV-2 S gene (GenBank: MN908947.3), S1 subdomain (aa 14–685), RBD (aa 331–524), S1-∆RBD (aa 14–330 and 525–685), SARS-CoV S gene (GenBank: AAP13441.1), and MERS-CoV S gene (GenBank: KF186567.1) were cloned into the pCAGGS-Myc vector and confirmed by sequencing analysis.

### Virus infection assay

For HRB25 infection, cells were infected at the indicated time and MOI for 1 h at 37 °C. The cells were then washed three times with 2% FBS-containing medium, and 2% FBS-containing medium was added to them. HEK293 cells (1.5 × 10^5^ cells) or HEK293-ACE2 cells (1.5 × 10^5^ cells) were seeded onto 24-well plates for 16 h. Then, the cells were transfected with mGluR2-Flag plasmid for 48 h, and a viral infection assay was performed (MOI = 0.1). We used the MOI determined in Vero-E6 cells as the standard relative dose.

### Flow cytometry

To detect the expression of mGluR2 or ACE2, Vero-E6 cells, Caco-2 cells, HEK293 cells, or HEK293-ACE2 cells were seeded onto 6-well plates. After 12 h, the cells were trypsinized with 0.25% trypsin (without EDTA) to harvest the cells. HPAE cells were seeded in 48-well plates for 72 h, then treated as described above. To detect mGluR2 and ACE2 on the cell membrane after virus infection, Vero-E6 cells (8 × 10^5^ cells/well) were seeded onto 6-well plates for 16 h and then infected with HRB25 (4 × 10^6^ PFU/well) in a volume of 1 mL at 4 °C for 1 h or at 37 °C for 30 min. The cells were then harvested as described above. To detect the expression of mGluR2 or ACE2 on mGluR2-silenced cell membranes, Vero-E6 cells or Caco-2 cells were transfected with mGluR2 siRNA for 72 h, and then the cells were harvested for flow cytometric analysis. The cells were fixed with 3% paraformaldehyde at room temperature for 15 min, then washed three times with FACS wash buffer (PBS containing 2% FCS), and incubated for 1 h with mGluR2 antibody (Santa Cruz Biotechnology, sc-271654), ACE2 antibody (R&D system, AF933), IgG2a isotype antibody (Southern Biotech, 0103-01), or IgG isotype antibody (Abcam, ab37373), which served as a control. They were then washed and stained with goat anti-mouse Alexa Fluor 488 (Thermo Fisher, A11034) or donkey anti-goat Alexa Fluor 488 (Abcam, ab150129) for 1 h. All cells were analyzed by using a FC500 flow cytometer (Beckman Coulter). Cell surface mean fluorescence density was measured and analyzed by using FlowJo software (FlowJo LLC).

### RNAi

siRNA transfections were performed by using the Lipofectamine RNAiMAX transfection reagent (Thermo Fisher Scientific, 13778150) according to the manufacturer’s instructions. Briefly, siRNA (1 μM, 40 μL per well, African green monkey, or Sigma 1 μM, 30 μL per well, human, ambion) targeting the mGluR2, ACE2, or non-targeting siRNA, was mixed with OptiMEM medium containing 0.8 μL of Lipofectamine RNAiMAX transfection reagent in a volume of 120 μL per well on 24-well plates. After a 30-min incubation at room temperature, Vero-E6 cells (6.7 × 10^4^ cells/well) and Caco-2 cells (5.4 × 10^4^ cells/well) were seeded into siRNA-coated 24-well plates in a volume of 500 μL per well. The RNAi assay of HPAE cells (5 × 10^4^ cells/well) was performed with siRNA (1 μM, 20 μL per well, human, ambion) mixed with 80 μL of OptiMEM medium containing 0.8 μL of RNAiMAX in 48-well plates. At 18 h after the siRNA transfection, mGluR2 mRNA or ACE2 mRNA was assessed by use of qPCR. At 72 h post-transfection, the cells were infected with HRB25 (MOI = 0.01 for Vero-E6 cells; MOI = 0.05 for Caco-2 cells; MOI = 0.001 for HPAE cells) for further studies. The siRNA sequences were as follows: simGluR2 #1, Sense: 5′-CAUUGAGGCCUUUGAGCUAdTdT-3′, Antisense: 5′-UAGCUCAAAGGCCUCAAUGdTdT-3′; simGluR2 #2, Sense: 5′-CGAUUGGACGAAUUCACUUtt-3′, Antisense: 5′-AAGUGAAUUCGUCCAAUCGgt-3′; siACE2, Sense: 5′-GCAUAUGCUGCACAACCUUdTdT-3′, Antisense: 5′-AAGGUUGUGCAGCAUAUGCdTdT-3′.

### Plaque assay

Viral titers from the cell culture medium or animal tissues were determined by the use of PFU assays. Serial dilutions of supernatants from infected cells or animal tissue homogenates were added to Vero-E6 cell monolayer and adsorbed for 1 h at 37 °C. Cells were then washed and plaque media was overlaid onto the cells, which were then placed back at 37 °C. After 48 h of incubation, the cell monolayers were stained with crystal violet and plaques were counted.

### TCID_50_ assay

Briefly, serial 10-fold dilutions of virus supernatant were made and 100 μL of each dilution was added to Vero-E6 cells in quadruplicate in 96-well plates. The plates were incubated for 24 h at 37 °C and then the GFP-expressing cells were observed under a fluorescence microscope. The 50% tissue culture infective dose (TCID_50_) was calculated by using the method of Reed & Muench.

### qPCR

The viral RNA copies in the samples collected from animals were determined as described previously^45^. Briefly, viral RNA was extracted by using a QIAamp vRNA Minikit (Qiagen) and reverse transcription was performed by using the HiScript® II Q RT SuperMix for qPCR (Vazyme). qPCR was conducted by using the Applied Biosystems® QuantStudio® 5 Real-Time PCR System (Thermo Fisher) with Premix Ex Taq™ (Probe qPCR), Bulk (TaRaKa), and SARS-CoV-2 N gene-specific primers (F: 5′-GGGGAACTTCTCCTGCTAGAAT-3′; R: 5′-CAGACATTTTGCTCTCAAGCTG-3′) and a probe (5′-FAM-TTGCTGCTGCTTGACAGATT-TAMRA-3′). The amount of viral RNA for the target SARS-CoV-2 N gene was normalized to a standard curve obtained by using a plasmid (pBluescriptIISK-N, 4221 bp) containing the full-length cDNA of the SARS-CoV-2 N gene.

To detect mGluR2 mRNA, ACE2 mRNA, and viral RNA in cells, total RNA from cells was isolated using TRIZOL reagent (Thermo Fisher) and was reverse-transcribed by using the Easyscript First-Strand cDNA synthesis Supermix (Transgen, AE301) according to the manufacturer’s instructions. Relative mRNA expression was analyzed by using SYBR green qPCR Master Mix (Vazyme) with the indicated mGluR2, ACE2, SARS-CoV-2 N, and VSV (Indiana strain) P gene-specific primers. The 2^−∆∆Ct^ method was used to calculate the relative gene expression level, with β-actin (Vero-E6 cells) or 28S rRNA (Caco-2 cells, HPAE cells) as the internal control. The sequences of the primers synthesized for qPCR were as follows: mGluR2-human, F: 5′-GCACAGGCAAGGAGACAGC-3′, R: 5′-GAGGCAGCCAAGCACCAC-3′; mGluR2-green monkey, F: 5′-GCTACAACATCTTCACCTA-3′, R: 5′-CACACTCTTCACCTCATT-3′; ACE2-human, F: 5′-GGTCTTCTGTCACCCGATTT-3′, R: 5′-CATCCACCTCCACTTCTCTAAC-3′; ACE2-green monkey, F: 5′-TGGGACTCTGCCATTTACTTAC-3′, R: 5′-CCCAACTATCTCTCGCTTCATC-3′; VSV (Indiana strain) P gene, F: 5′-GTGACGGACGAATGTCTCATAA-3′, R: 5′-TTTGACTCTCGCCTGATTGTAC-3′; 28S rRNA-human, F: 5′-GGGTGGTAAACTCCATCTAAGG-3′, R: 5′-GCCCTCTTGAACTCTCTCTTC-3′; and β-actin-green monkey, F: 5′-GACAGGATGCAGAAGGAGATTAC-3′, R: 5′-CTGCTTGCTGATCCACATCT-3′.

### Western blot analysis

The supernatant of cell lysates was diluted in denaturing buffer and boiled for 15 min. After denaturing, the samples were loaded onto a 4%–12% SDS-PAGE gel (Genscript) and separated by electrophoresis. Proteins were transferred to a PVDF membrane (Merck-Millipore, ISEQ00010). The PVDF membrane was blocked with 5% skim milk in PBS containing 0.1% Tween-20, and then incubated with the primary antibodies: anti-Flag antibody (1:1000, Genscript, A00187), anti-Myc antibody (1:1000, Genscript, A00172), or anti-GST antibody (1:1000, Genscript, A00097). Then, the membrane was washed three times with PBS and incubated with HRP-conjugated goat anti-mouse antibody (1:10,000, Genscript, A00160) and goat anti-rabbit antibody (1:10,000, Genscript, A00098). After three washes with PBST buffer, target protein bands were detected by using the enhanced chemiluminescence (ECL) reagent (Merck Millipore, WBLUR0500).

### Co-immunoprecipitation

mGluR2-Flag and SARS-CoV-2 S-Myc, SARS-CoV-2 S1-Myc, SARS-CoV-2 RBD-Myc, SARS-CoV-2 S1-∆RBD-Myc, SARS-CoV S-Myc, ACE2-Myc, MERS-CoV S-Myc, and DPP4-Myc were respectively co-transfected into HEK293 cells with TransIT-293 transfection reagent (Mirus, MIR2701) by following the manufacturer’s instructions. At 48 h post-transfection, the cells were washed with PBS and lysed with 1% NP-40 buffer (Beyotime, P0013F) containing a protease inhibitor for 1 h at 4 °C. Cell lysates were centrifuged (12,000 rpm) for 20 min at 4 °C to remove cell debris. Then, the supernatant was collected and mixed with 40 μL of protein G Agarose (Roche, 11243233001) for 4 h at 4 °C on a flip shaker. The protein G beads were then removed by centrifugation, and the supernatant was collected and mixed with anti-Flag antibody-conjugated agarose beads (Sigma, A2220) for 6 h at 4 °C on a flip shaker. After conjugation, the beads were washed five times with pre-chilled 1% NP-40 PBS buffer. Finally, the beads were resuspended in PBS and mixed with protein sample loading buffer, boiled for 15 min, and subjected to SDS-PAGE.

### Pull-down assay

For pull-down assays, the N-terminal GST-tagged soluble ectodomain of mGluR2 (mGluR2-GST, aa 19–567) was expressed and purified by FriendBio Technology (Wuhan, Hubei, China). The soluble SARS-CoV-2 S1 protein (SARS-CoV-2 S1-His, aa 14–681) was expressed in transfected BHK-21 cells and purified for the pull-down assay. GST protein was used as the negative control. The purified GST-tagged proteins were incubated with Glutathione Sepharose 4B beads (GE Healthcare Bio-science, 17-0756-01) at 4 °C for 2 h. The beads were then washed and incubated with whole-cell lysates from HEK293 cells expressing Myc-tagged proteins, the soluble SARS-CoV-2 S1-His protein (10 μg), or the lysates from SARS-CoV-2-infected Vero-E6 cells at 4 °C for 5 h with constant rotation. After conjugation, the beads were washed five times with wash buffer (pH 8.5, 20 mM Tris, 500 mM NaCl, 2 mM EDTA) and re-suspended in PBS and protein sample loading buffer. The samples were then subjected to SDS-PAGE, and assessed by western blot analysis.

### Cell viability assay

Cell viability was determined by using the Cell Titer-Glo kit (Promega, G9242). Cells were seeded onto 96-well plates with opaque walls. Antibody at the indicated concentrations was added, and 48 h later, Cell Titer-Glo reagent was added to each well. Luminescence was measured with a GloMax 96 Microplate Luminometer (Promega).

### Antibody blocking assay

Vero-E6 cells (4 × 10^4^ cells/well) or Caco-2 cells (4 × 10^4^ cells/well) were seeded onto 96-well plates for 16 h. Cells were treated with the indicated concentrations of mGluR2 antibody (Santa Cruz Biotechnology, sc-271654), or isotype antibody (Southern Biotech, 0103-01), ACE2 antibody (R&D system, AF933), or isotype antibody (R&D system, AB-108-C) for 1 h on ice. Vero-E6 cells (MOI = 0.002) and Caco-2 cells (MOI = 0.02) were infected with HRB25, for 1 h at 4 °C in the presence of the indicated concentrations of antibody, then washed, and incubated with medium containing antibody at 37 °C. At 48 h p.i., the culture supernatant was harvested to assess virus titers by use of the plaque-forming assay.

HPAE cells (5 × 10^4^ cells/well) were seeded onto 48-well plates for 72 h, then treated with mGluR2 antibody (15 μg/mL) or isotype antibody (15 μg/mL), and infected with HRB25 (MOI = 0.01) as described above. At 48 h p.i., the viral RNA expression level relative to 28S rRNA was calculated by qPCR.

Vero-E6 cells were treated with the indicated concentrations of mGluR2 antibody, or isotype antibody, then infected with VSV (MOI = 0.005), rVSV-SARS-CoV-S (MOI = 0.05), or rVSV-MERS-CoV-S (MOI = 0.05) as described above. The infection culture supernatant of VSV was harvested at 24 h p.i.

### Soluble mGluR2 ectodomain neutralization assay

Vero-E6 cells (2 × 10^5^ cells/well) were seeded onto 24-well plates for 16 h. HRB25 (MOI = 0.0002) was mixed with the purified N-terminal GST-tagged soluble ectodomain of mGluR2 at different concentrations or with GST in 100 μL of cell culture medium at 4 °C for 1 h. Cells were then incubated with the virus-protein mix at 37 °C for 1 h, then washed and incubated with growth medium. At 24 h p.i., the culture supernatant was harvested to assess virus titers by plaque-forming assay.

### Viral binding assay

Cell-bound HRB25, rVSV-SARS-CoV-S, or rVSV-MERS-CoV-S was assessed by use of qPCR. Cells were transfected with the indicated siRNA for 72 h, then the cells were transferred onto ice for 20 min. Then, 200 μL of HRB25 (2 × 10^6^ PFU/well), rVSV-SARS-CoV-S (2 × 10^6^ PFU/well), or rVSV-MERS-CoV-S (2 × 10^6^ PFU/well) was added to the cells at 4 °C for 1 h. Unbound virions were removed by three washes with pre-chilled PBS, and the cells were then lysed by TRIZOL. The viral RNA expression level relative to β-actin (Vero-E6 cells) or 28S rRNA (Caco-2 cells) was then calculated by use of qPCR.

### Viral internalization assay

Internalized virions were detected by the use of qPCR. Cells were transfected with the indicated siRNA for 72 h and then were transferred onto ice for 20 min. HRB25 (2 × 10^6^ PFU/well), rVSV-SARS-CoV-S (2 × 10^6^ PFU/well), or rVSV-MERS-CoV-S (2 × 10^6^ PFU/well) was then added to cells at 4 °C for 1 h. After removal of unbound virions by extensive washing with chilled PBS, the cells were moved to 37 °C for 1 h to allow internalization. After 1 h, the cells were washed three times for 3 min with acidic buffer (50 mM glycine, 100 mM NaCl, pH 3.0), then trypsinized to remove HRB25, rVSV-SARS-CoV-S, or rVSV-MERS-CoV-S bound to the cell surface. The cells were lysed for total RNA extraction and subjected to qPCR to detect internalized viruses.

For microscopy, Vero-E6 cells were transfected with the indicated siRNA and cultured on Millicell EZ slide 4-Well Glass (Merck Millipore, PEZGS0416) for 72 h. After HRB25 internalization for 1 h at 37 °C, the cells were immediately fixed with 3% paraformaldehyde for 15 min at room temperature. If needed, they were permeabilized with 0.1% Triton X-100 in PBS for 10 min at room temperature and then incubated with 1% BSA for 30 min to block non-specific binding of antibodies. Both permeabilized and unpermeabilized cells were incubated with anti-SARS-CoV-2 S protein rabbit monoclonal antibody (1:100, Sino Biologicals, 40150-R007) overnight at 4 °C, then washed and stained with goat anti-rabbit Alexa Fluor 488 (1:1000, Thermo Fisher, A11034) for 1 h. Nuclei were visualized by staining with Hoechst 33342, and coverslips were mounted in Fluoroshield™ histology mounting medium (Sigma, F6182) onto slides. Fluorescence intensity was quantified with a Zeiss LSM880 laser-scanning confocal microscope (Carl Zeiss AG) equipped with Airyscan (Plan-Apochromat, objective 63×, 1.4 Numerical Aperture DIC oil immersion objective) by using ZEN software. The resolution of the acquired images was 1024 × 1024. The cell-bound HRB25 signal intensities from at least 110 cells per sample were quantified by using ZEN software.

### Inhibitor assay

Caco-2 cells were plated in 24-well plates, transfected with scrambled siRNA or mGluR2 siRNA for 72 h, and then preincubated with DMSO, E64D (MCE, HY-100229, 100 μM), or camostat mesylate (MCE, HY-13512, 300 μM) for 2 h at 37 °C. The virus infection assay was then performed. Caco-2 cells were infected with HRB25 (MOI = 1) for 1 h at 37 °C. After the cells were washed, medium contained the indicated inhibitors were added. Viral RNA levels in the cell lysate relative to 28S rRNA was measured by use of qPCR at 8 h p.i. and 16 h p.i..

### Multiplex immunofluorescence

Vero-E6 cells (1 × 10^5^ cells/well) or HPAE cells (5 × 10^4^ cells/well) were cultured on Millicell EZ slide 4-Well Glass, and infected with 200 μL of HRB25 (5 × 10^6^ PFU/well for Vero-E6 cells, 5 × 10^5^ PFU/well for HPAE cells) at 37 °C for 5 min. Then, the cells were thoroughly washed with PBS and fixed with 3% paraformaldehyde. Multiplex immunofluorescence with Tyramide Signal Amplification (TSA) was performed by following the previously established protocol^29^. Briefly, endogenous peroxidase activity was quenched. After permeabilization with 0.1% Triton X-100 and blocking steps (Zsbio, ZLI-9056), the samples were incubated with primary antibodies followed by HRP-conjugated secondary antibodies. Multiplex fluorescence labeling was performed using TSA-dendron-fluorophores (NEON 7-color Allround Discovery Kit for FFPE, Histova Biotechnology, NEFP750). A commercial antibody stripping buffer was employed to remove the primary and secondary antibodies while retaining the TSA signal by incubation for 30 min at 37 °C. After a brief rinse, other antigens were serially detected by using spectrally different TSA reagents and following the above method. The primary antibodies used in this study were: ACE2 (1:400, Abcam, ab108252), SARS-CoV-2 nucleocapsid protein (1:1000, Sinobiological, 40143-R004), mGluR2 (1:200, Santa Cruz Biotechnology, sc-271654), and clathrin (1:400, CST, 4796S). The secondary antibodies were: HRP-conjugated anti-rabbit IgG (Zsbio, PV-6001) and HRP-conjugated anti-mouse IgG (Zsbio, PV-6002). Images were acquired by using a Zeiss LSM880 laser-scanning confocal microscope equipped with Airyscan. The resolution of the acquired images was 2048 × 2048.

Data were processed using Bitplane Imaris software (Bitplane AG, Zurich, Switzerland) by following the established protocol^29^. Briefly, first, the red channel (SARS-CoV-2) and purple channel (clathrin or ACE2) were processed using the “surface module”. Then, the surface results of the purple and red channels were inputted as “cell” and “nuclei”, respectively, under the “cell module”. Next, the green channel (mGluR2) was processed using the “surface module”. After that, a new channel was established by merging the red and green channels using the “mask dataset” of the “coloc” module. Spots that represented the co-localization of SARS-CoV-2 and mGluR2 were counted in the merged channel by using the “spot” module. Finally, the spot results were inputted into “cell”, and the SARS-CoV-2-mGluR2 spots that co-localized with clathrin or ACE2 were counted.

Multiplex immunofluorescence staining for the detection of SARS-CoV-2-targeted cells in the olfactory epithelium and mouse lung sections was performed. Briefly, 4-μm-thick paraffin sections were deparaffinized in xylene and rehydrated in a series of graded alcohols. Antigen retrievals were performed in citrate buffer (pH 6.0) by using a microwave oven for 20 min at 95 °C followed by a 20-min cooldown at room temperature. Multiplex fluorescence labeling was performed using TSA-dendron-fluorophores (NEON 7-color Allround Discovery Kit for FFPE, Histova Biotechnology, NEFP750). Briefly, primary antibody was incubated for 2–4 h in a humidified chamber at 37 °C, followed by detection using the HRP-conjugated secondary antibody and TSA-dendron-fluorophores. Afterwards, the primary and secondary antibodies were thoroughly eliminated by heating the slides in retrieval/elution buffer (Abcracker, Histova Biotechnology, ABCFR5L) for 10 s at 95 °C in a microwave. In serial fashion, each antigen was labeled with distinct fluorophores. Multiplex immunofluorescence staining of a normal human (male, 21 years old) lung section, which was obtained from Shanghai Biochip Company, China, was performed as described above.

The multiplex antibody panels applied in this study were: mGluR2 (1:400, Abcam, ab150387), Ace2 (1:400, Abcam, ab108252), SARS-CoV-2 nucleocapsid protein (1:1000, Sinobiological, 40143-R004), GAP43 (1:1000, Abcam, ab75810), OMP (1:1500, Abcam, ab183947), CK5 (1:800, Abcam, ab52635), CK8 (1:800, Abcam, ab53280), CC10 (1:500, Millipore, 07-623), Foxj1 (1:1000, Abcam, ab235445), SPC (1:500, Abcam, ab211326), and Tubb4 (1:1000, Abcam, ab179504). After all the antibodies were detected sequentially, the slices were imaged using the confocal laser scanning microscopy platform Zeiss LSM880.

### Genotypic identification

The genotype of the mice was identified by using a PCR assay. Tail tissues were obtained from 6-week-old mice and whole-genome DNA was extracted with the TIANamp Genomic DNA Kit (TIANGEN DP304-03) by following the manufacturer’s instructions. WT mGluR2 was identified with specific primers (F, 5′-CGTGGCCTGATATCTCTACCGT-3′; R, 5′-TGTCCACAGTGTGGTGCTGAAT-3′) and mGluR2^−/−^ was identified with specific primers (F, 5′-CGTGGCCTGATATCTCTACCGT-3′; R, 5′-ACCGTCTCCTAGAAGAGTGGACA-3′). 2× Taq reaction component (Vazyme, P112-03) was used in the reaction. The conditions for PCR were as follows: one cycle of 95 °C for 5 min, 20 cycles of 98 °C for 30 s, 65 °C for 30 s and drop 0.5 °C per cycle, 72 °C for 45 s, 20 cycles of 98 °C for 30 s, 55 °C for 30 s, 72 °C for 45 s, and one cycle of 72 °C for 5 min.

### Animal experiments

WT and mGluR2^*−/−*^ mice were inoculated intranasally with 150 PFU of HRB26M in a volume of 50 μL^33^. At 3 days p.i., all mice in each group were euthanized and their nasal turbinates and lungs were collected to detect viral RNA and infectious viruses. Viral RNA copies and infectious titers were detected by use of qPCR and plaque assays, respectively.

### EGF internalization assay

For the EGF internalization assay, mGluR2-silenced Vero-E6 cells were washed twice with PBS and maintained in serum-free DMEM for 2 h at 37 °C. Then, the cells were washed with PBS and incubated with live cell imaging solution (Invitrogen, A14291DJ, 20 mM glucose, 1% BSA) containing 2 µg/mL Alexa Fluor 647-labeled EGF (Invitrogen, E35351) for 40 min. To stop the internalization, the cells were washed twice with cold PBS and then incubated twice for 2 min in cold wash buffer (50 mM glycine, 100 mM NaCl, pH 3.0). After removing the unbound EGF, the cells were harvest for flow cytometric analysis.

## Supplementary information

Supplementary information

## Data Availability

All data are available in the main text or supplementary materials. Sequences of the viruses used in this study have been deposited in GISAID (accession numbers EPI_ISL_467430 and EPI_ISL_459910).
