# Supplementary material for: SARS-CoV-2 uses metabotropic glutamate receptor subtype 2 as an internalization factor to infect cells
Source: Cell Discov. 2021 Dec 14;7:119. doi: 10.1038/s41421-021-00357-z (PMC8668938; doi:10.1038/s41421-021-00357-z)
Supplement: Supplementary file 1 — Supplementary information [file 41421_2021_357_MOESM1_ESM.pdf]

Supplementary Information

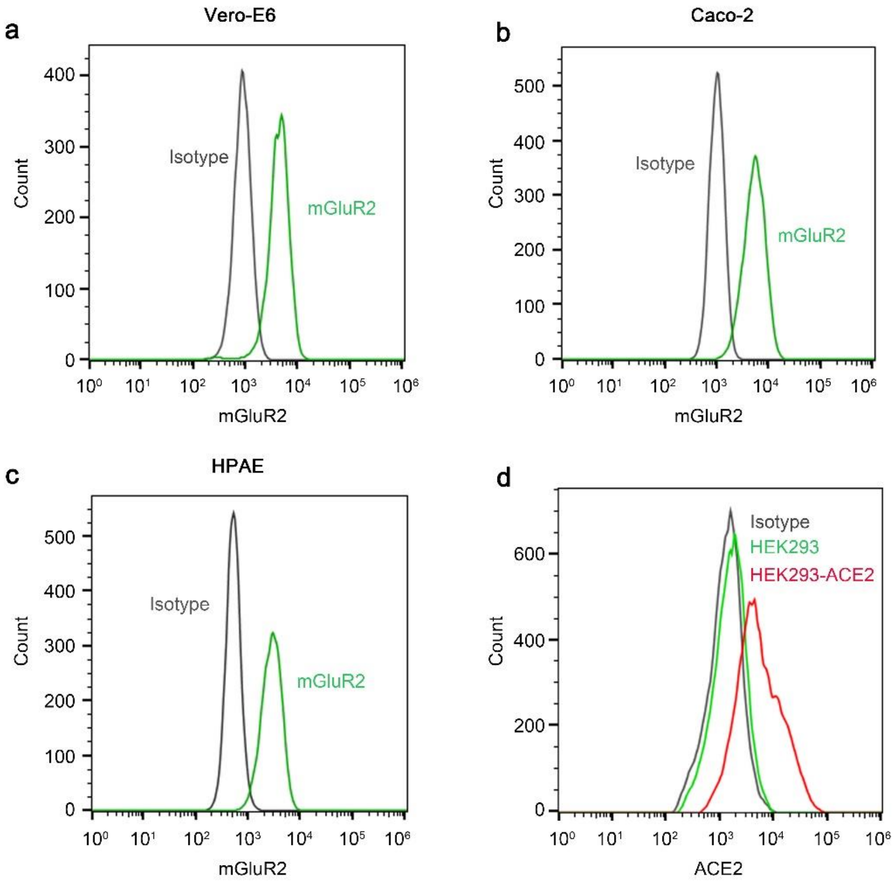

Supplementary Fig. S1 Expression of mGluR2 or ACE2 on Vero-E6 cells, Caco-2 cells, HPAE cells, HEK293 cells, or HEK293-ACE2 cells. a-c Surface expression of mGluR2 on Vero-E6 cells (**a**), Caco-2 cells (**b**) and HPAE cells (**c**) was confirmed by flow cytometry. **d** Surface expression of ACE2 on HEK293 cells or HEK293-ACE2 cells was detected by flow cytometry.

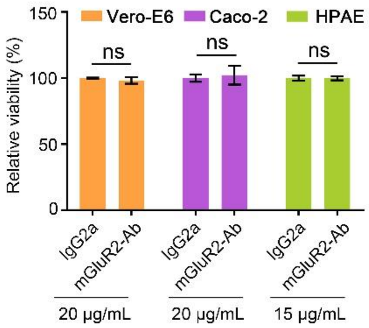

Supplementary Fig. S2 Cell viability is unaffected by mGluR2 antibody treatment. Vero-E6 cells, Caco-2 cells, or HPAE cells were treated with mGluR2 antibody at different concentrations or IgG2a (20 μg/mL for Vero-E6 cells and Caco-2 cells; 15 μg/mL for HPAE cells) for 48 h at 37 °C. Then cell viability was determined by using a commercial cell viability assay kit. The data shown are representative results from three independent experiments (n = 3), mean ± SD, Student's test, ns, not significant.

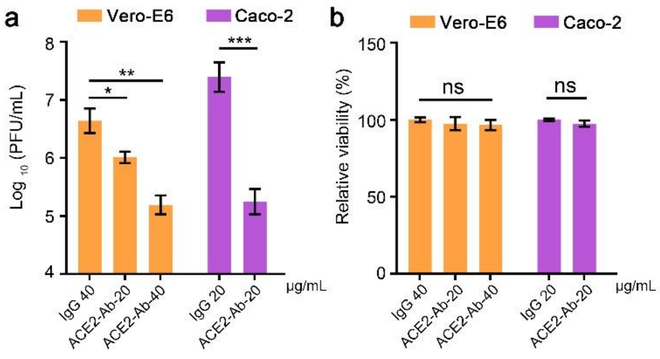

Supplementary Fig. S3 An antibody against ACE2 blocks SARS-CoV-2 infection in cells. **a** Vero-E6 cells and Caco-2 cells were treated with ACE2-Ab or IgG at different concentrations for 1 h at 4 °C, then infected with HRB25. Virus in the culture supernatant was detected by use of plaque assays at 48 h p.i.. **b** Cell viability was determined by using a commercial cell viability assay kit. The data shown are representative results from three independent experiments (n = 3), mean ± SD, Student's test, ns, not significant, $^*P < 0.05$, $^{**}P < 0.01$, $^{***}P < 0.001$.

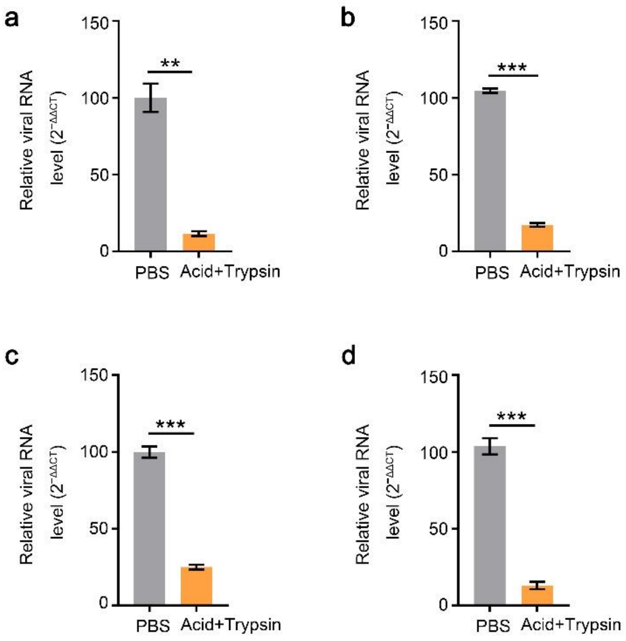

Supplementary Fig. S4 Cells treated with acid buffer/trypsin could efficiently remove bound SARS-CoV-2, rVSV-SARS-CoV-S, or rVSV-MERS-CoV-S. a, b Vero-E6 cells (**a**) and Caco-2 cells (**b**) were incubated with HRB25 for 1 h at 4 °C and washed to remove unbound virus. The cells were washed with acid buffer/trypsin and lysed for qPCR to detect SARS-CoV-2 bound to the cell surface. **c, d** Vero-E6 cells were treated as described in (**a**), but incubated with rVSV-SARS-CoV-S (**c**) or rVSV-MERS-CoV-S (**d**). The data shown are representative results from three independent experiments (n = 3), mean ± SD, Student's test, $^{**}P < 0.01$, $^{***}P < 0.001$.

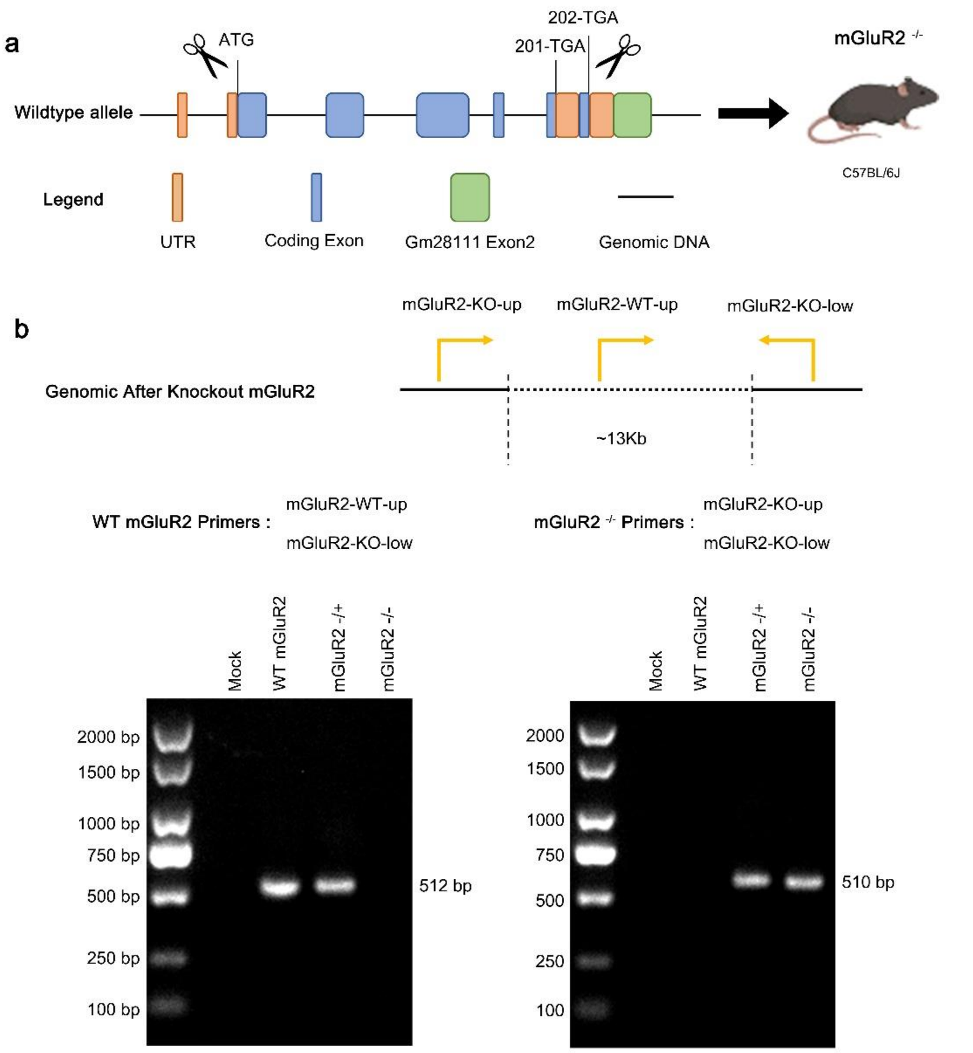

Supplementary Fig. S5 Generation of mGluR2 gene knockout mouse. a Diagram of the mGluR2 gene deletion. **b** Genotypic identification with PCR analysis of genomic DNA samples obtained from mouse tails.

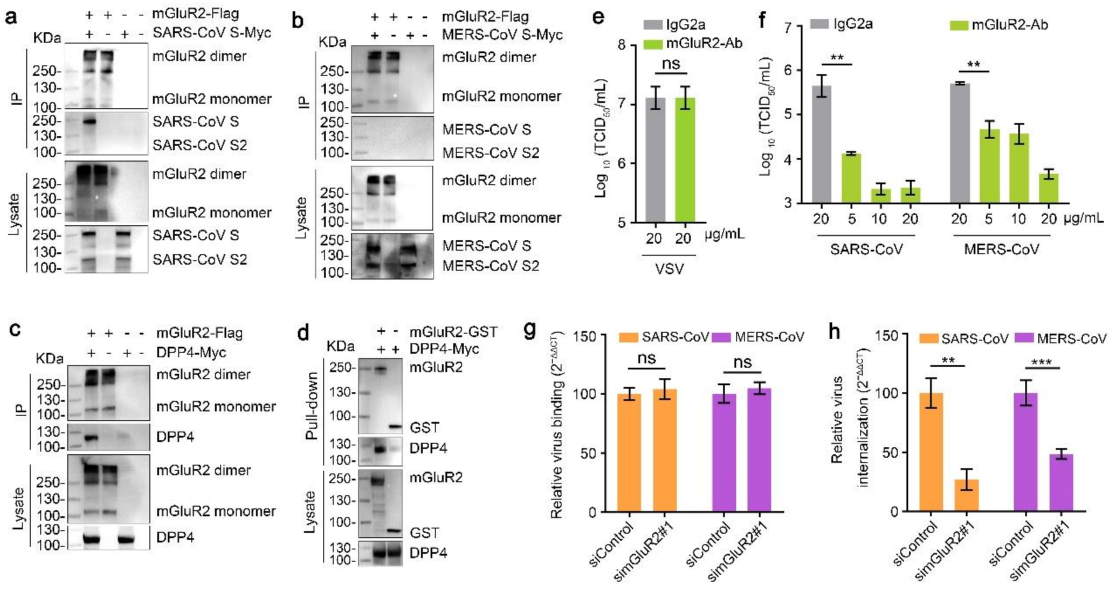

Supplementary Fig. S6 mGluR2 is important for SARS-CoV S- and MERS-CoV S-mediated internalization. a-c mGluR2-Flag and SARS-CoV S-Myc (**a**), MERS-CoV S-Myc (**b**), or DPP4-Myc (**c**) were co-transfected into HEK293 cells, and were then immunoprecipitated by using anti-Flag agarose beads. **d** mGluR2-GST was pooled with lysate from DPP4-Myc-transfected HEK293 cells and then pulled-down by using anti-GST beads. **e, f** Vero-E6 cells were treated with mGluR2-Ab, or IgG2a (20 μg/mL) for 1 h at 4 °C, then infected with VSV (**e**), rVSV-SARS-CoV-S, or rVSV-MERS-CoV-S (**f**) for 1 h at 4 °C. Virus in the culture supernatant was detected by using $TCID_{50}$ assays at 24 h p.i.. **g, h** Viral binding (**g**) and internalization assays (**h**) were performed in mGluR2-silenced Vero-E6 cells. Cells were incubated with rVSV-SARS-CoV-S or rVSV-MERS-CoV-S, respectively. The data shown are representative results from three independent experiments (**e-h**, n = 3), mean ± SD, Student's test, ns, not significant, $**P < 0.01$, $***P < 0.001$.

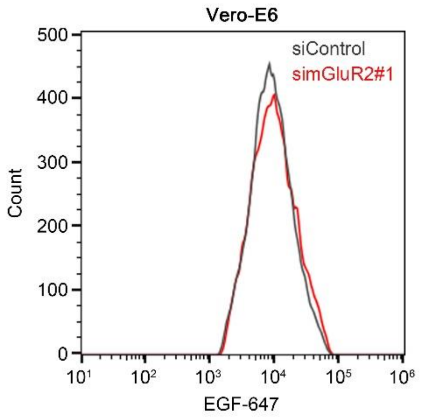

Supplementary Fig. S7 Knockdown of mGluR2 has no effect on EGF uptake. mGluR2-silenced Vero-E6 cells were serum-starved and then incubated with 2 µg/mL Alexa647-labeled EGF for 40 min at 37 °C. The fluorescence signal was detected by use of flow cytometry.

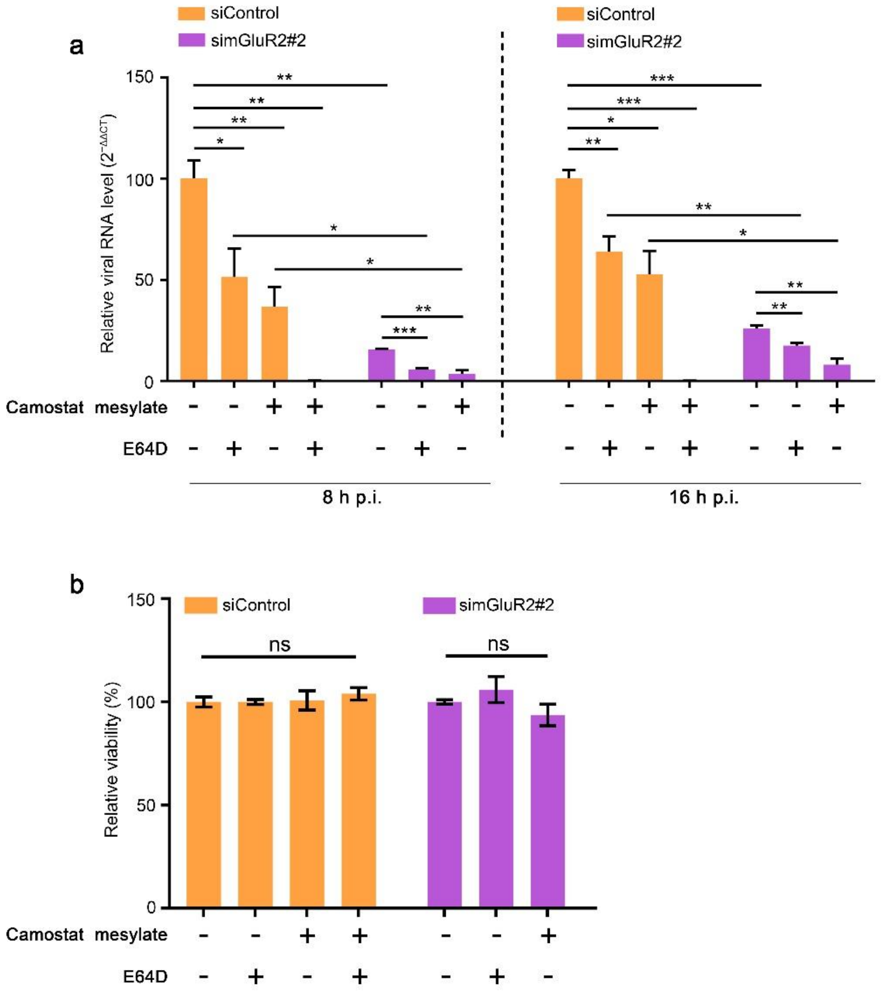

Supplementary Fig. S8 mGluR2 affects direct fusion during SARS-CoV-2 infection. a Caco-2 cells were preincubated with DMSO, E64D (100 µM), or camostat mesylate (300 µM) for 2 h at 37 °C, and then infected with HRB25 (MOI=1). The viral RNA level in the cell lysate was measured by use of qPCR at 8 h and 16 h p.i., respectively. **b** scrambled siRNA-transfected Caco-2 cells or mGluR2-silenced Caco-2 cells were incubated with DMSO, E64D (100 µM), or camostat mesylate (300 µM) for 16 h at 37 °C. Then, cell viability was determined by using a commercial cell viability assay kit. The data shown are representative results from three independent experiments (n = 3), mean ± SD, Student's test, ns, not significant, **P* < 0.05, ***P* < 0.01, ****P* < 0.001.
